# Use of Fluorescence Lifetime Imaging Microscopy (FLIM) as a Timer of Cell Cycle S Phase

**DOI:** 10.1371/journal.pone.0167385

**Published:** 2016-12-14

**Authors:** Irina A. Okkelman, Ruslan I. Dmitriev, Tara Foley, Dmitri B. Papkovsky

**Affiliations:** 1 School of Biochemistry and Cell Biology, ABCRF, University College Cork, College Road, Cork, Ireland; 2 Department of Anatomy and Neuroscience, University College Cork, Western Road, Cork, Ireland; The Francis Crick Institute, UNITED KINGDOM

## Abstract

Incorporation of thymidine analogues in replicating DNA, coupled with antibody and fluorophore staining, allows analysis of cell proliferation, but is currently limited to monolayer cultures, fixed cells and end-point assays. We describe a simple microscopy imaging method for live real-time analysis of cell proliferation, S phase progression over several division cycles, effects of anti-proliferative drugs and other applications. It is based on the prominent (~ 1.7-fold) quenching of fluorescence lifetime of a common cell-permeable nuclear stain, Hoechst 33342 upon the incorporation of 5-bromo-2’-deoxyuridine (BrdU) in genomic DNA and detection by fluorescence lifetime imaging microscopy (FLIM). We show that quantitative and accurate FLIM technique allows high-content, multi-parametric dynamic analyses, far superior to the intensity-based imaging. We demonstrate its uses with monolayer cell cultures, complex 3D tissue models of tumor cell spheroids and intestinal organoids, and in physiological study with metformin treatment.

## Introduction

Analysis of cell proliferation is essential for studies of cellular function, effects of drugs, various biological factors and treatments. Classical methods of analysis of cell proliferation are based on incorporation of thymidine analogues during DNA replication and/ or labeling with a suitable tracers such as ^3^H-thymidine, fluorescent antibody or dye reacting with 5-bromo-2’-deoxyuridine (BrdU) or 5-ethynyl-2’-deoxyuridine, respectively [1–3]. Fluorescence-based microscopy and flow cytometry platforms have replaced the unsafe autoradiography [4, 5], but they still remain tedious, mostly end-point, suffer from antibody variability, the need of epitope unmasking, limited in-depth staining and toxicity of click-reaction products. The use of transiently or stably expressed genetically encoded fluorescent proteins fused with cell cycle markers is also complex, can influence cell cycle, and have limited use with primary cells and complex 3D models [6, 7].

Hoechst are a family of cell-permeable bis-benzimide dyes, which bind to the minor groove of double-stranded (ds) DNA with strong enhancement of their blue fluorescence and bright staining of cell nuclei. BrdU incorporated in dsDNA was seen to quench Hoechst 33342 (HXT) and Hoechst 33358 fluorescence via heavy atom effect [8]. This was proposed to use for detection of proliferation by flow cytometry of fixed or live cells [9–12]. However high variability of fluorescence intensity signals (depend on fluorophore concentration, size of the nuclei, cell shape and photobleaching) prevented widespread use of this approach [13].

In contrast, fluorescence lifetime, being a structural and environmental signature of a fluorophore dye [13, 14], is largely independent on the above interfering factors. Fluorescence Lifetime Imaging Microscopy (FLIM) allows discrimination of fluorophors with different structures, lifetime characteristics and microenvironment, and is well-suited for quantitative, multi-parametric imaging of complex biological specimens [15]. Development of FLIM hardware such as time-correlated single photon counting (TCSPC) and dedicated fluorescent and phosphorescent probes have prompted their broad use in live imaging of cellular autofluorescence and parameters such as pH, O_2_, T, Cl^-^ and Ca^2+^ [16–22]. However, no FLIM-based cell cycle assays based on microscopy have been described so far. Progress in regenerative medicine and biotechnology also calls for new assays to monitor proliferation and cell cycle progression in live cultures, especially 3D tissue, *ex vivo* and *in vivo* models [23, 24], and versatile FLIM techniques hold promise for such applications.

Here we describe a cell cycle assay based on BrdU and Hoechst 33342 (HXT) staining and FLIM measurement of live cells. We found that upon BrdU incorporation fluorescence lifetime of HXT markedly reduces, in time and concentration-dependent manner. We optimized this to enable simple and robust tracing of cell proliferation in culture, with accurate quantification of S phase duration and cell progression over several division cycles. The new method was demonstrated by monitoring dividing cells in multicellular tumor spheroids, amplification-transition zone of mouse intestinal organoids, and studying the effects of metformin drug on cell proliferation in the intestinal organoids.

## Methods

### Materials

CellTox Green Cytotoxicity Assay kit (G8742) was from Promega (MyBio, Ireland). Tetramethylrhodamine methyl ester (TMRM) (T-668), cholera toxin (CTX) subunit B Alexa Fluor 488 conjugate (C34775) and secondary Alexa Fluor 488-conjugated anti-mouse antibodies (A10680) were from Invitrogen (GE Healthcare, Ireland). Mouse monoclonal anti-BrdU antibody (clone BU-1, 05–633) was from Millipore (Cork, Ireland). Intesticult Organoid Growth Medium (mouse) kit (06005) and ‘gentle cell dissociation reagent’ (07174) were from Stem Cell Technologies (UK). Matrigel® with reduced growth factors (356231) was from Corning. Phosphorescent O_2_-sensitive probe Pt-Glc was synthesized as previously described [18]. Bis-benzimide Hoechst 33342 (B2261), 5-bromo-2’-deoxyuridine (B5002), aphidicolin from *Nigrospora sphaerica* (A4487), metformin hydrochloride (PHR1084), phosphate buffered saline (P4417), albumin from bovine serum (A4503), penicillin-streptomycin solution (P0781) and all the other reagents were from Sigma-Aldrich (Dublin, Ireland).

### Cell culture and intestinal organoid culture

MEF cells (ATCC, Manassas, VA) were cultured in high glucose DMEM supplemented with 10% FBS (heat-inactivated), 10 mM HEPES, pH 7.2, 2 mM l-glutamine. HCT116 cells (ATCC) were cultured in McCoy’s 5A media supplemented with 10% FBS, 10 mM HEPES, pH 7.2, 2 mM l-glutamine. Tumor spheroids were formed by seeding HCT116 cells on a Lipidure-coat^TM^ plate (Amsbio, UK) at concentration of 200 cells/ well, and by growing them for 4 days. For imaging, spheroids were transferred on glass-bottom 35 mm dishes (P35G-1.5-14-C, MatTek Corporation, USA) pre-coated with a mixture of 0.07 mg/ml collagen IV / 0.03 mg/ml poly-D-lysine, and allowed to attach for 2–3 h.

All the procedures with animals were performed under a licence issued by the Irish Government Department of Health and Children (Ireland) and in accordance with the Directive 2010/63/EU adopted by the European Parliament and the Council of the European Union. Mice were sacrificed using cervical dislocation, accordingly to the protocol, approved by Animal Experimentation Ethics Committee of University College Cork. Intestinal organoids were produced from intestinal crypts of female C57 Bl6/J mice as described in [25]. Approximately 500 crypts were seeded in 50 μl of Matrigel in 24-well plate (Corning) and cultured in Intesticult Organoid Growth Medium supplemented with growth factors. Splitting of organoid culture was done using Gentle Cell Dissociation Reagent according to the recommendations of manufacturer. For imaging, organoids were seeded in 20 μl of Matrigel in 35 mm tissue culture dishes (Sarstedt, 83.1800.003), inside the removable microchamber frames (Ibidi GmbH, Germany), and cultured for 6 days.

### Synchronization of cells by aphidicolin block

HCT116 cells were seeded at 50–75% confluence, allowed to attach for 3–4 h and then exposed to 1 μg/ml aphidicolin (APH) for 18 h. Synchronized cells were then washed three times with medium to remove the APH and start cell cycle from the S phase, with BrdU and HXT added, as described in Results section. For intestinal organoid cultures we used 0.5 μg/ml APH for 18 h.

### Live cell FLIM imaging of cell cycle

For imaging, growth medium was replaced with phenol red-free DMEM supplemented with 10 mM HEPES, pH 7.2, 1 mM sodium pyruvate, 10 mM D-glucose, 2 mM l-glutamine. In optimization experiments cells were incubated with variable concentrations of BrdU (100, 50, 25, 10, 5 μM) and HXT (4, 2, 1, 0.5 μM), for 4 h and 30 min, respectively. Incubation with HXT was done simultaneously with BrdU loading. For kinetic analysis of BrdU incorporation (100 or 25 μM BrdU), incubation with BrdU was done for 1, 2, 3, 4 and 6 h intervals 30 min after the release of APH block. For intestinal organoid culture 100 μM BrdU (4 h or 18 h) and 1.5 μM HXT (4 h) staining concentrations were used.

Other staining conditions were: Cholera toxin, subunit B, Alexa Fluor 488 conjugate (CTX, 1.25 μg/ml; 4 h), Pt-Glc (4 μM; 4 h), Cell Tox Green (0.1%, 4 h) and TMRM (20 nM, 0.5 h). Incubation was done with all probes simultaneously prior the imaging.

Fluorescence and phosphorescence lifetime imaging microscopy was performed on a standard FLIM-PLIM TCSPC system (Becker & Hickl GmbH, Germany) based on an upright AxioExaminer Z1 microscope (Carl Zeiss) with 20x/1.0 and 63x/1.0 W-Plan Apochromat dipping water immersion objectives, heated incubator and stage (T = 37°C) with motorized Z-axis control [26]. The microscope was connected to DCS-120 confocal scanner (Becker & Hickl) with two excitation and two emission channels, a 405 nm BDL-SMC picosecond diode laser (Becker & Hickl) and a picosecond supercontinuum 400–650 nm laser SC400-4 (Fianium, UK). Emission filters (Semrock) included: 438–458 nm (HXT), 635–675 nm (Pt-Glc), 512–536 nm (Alexa Fluor 488, Cell Tox Green) and 565–605 nm (TMRM). An R10467U-40 photon counting detector, with >30% quantum efficiency at 400–700 nm (Hamamatsu Photonics K.K.) was connected to the scanner and TCSPC hardware for emission detection. Routinely, the scanning was performed with 256x256 pixels resolution. MicroToolBox, version 2011 software (Carl Zeiss) controlled the microscope and image acquisition. Data processing was performed in SPCImage software (Becker & Hickl). Confocal microscopy with 3D spheroids was done with a step of 5 μm (15 optical sections). Immunofluorescence analysis was performed as described before [26].

### Data analysis and statistics

The initial fitting of fluorescence decays for HXT and phosphorescence decays for Pt-Glc and calculation of τ_m_ values were done in SPCImage software (Becker & Hickl), by adjusting the fitting parameters T_1_, T_2_ and binning factor for randomly selected pixels in the images. For HXT we used the double-exponential function and the following initial settings: T_1_ = 31, T_2_ = 240, binning—5, shift—0, scatter—0, channel 25, ‘tail enhanced fit’, and multiple threads. These settings were applied to the whole field (256x256 pixels) to all HXT images. Threshold conditions were specified to the image to have zero background intensity level, which provided τ_m_ histograms of better quality and excluded areas without cells from analysis. For Pt-Glc τ_m_ were calculated from mono-exponential decay fits in SPCImage software with following settings: T_1_ = 84, T_2_ = 244, binning 7, threshold 40, shift 6.30, scatter 0, channel 22, ‘tail enhanced fit’, multiple threads. τ_m_ values were then converted into O_2_ concentration using simplified two-site model of Stern-Volmer equation [27]:

$$\frac{to}{t}=1/\left(\frac{f}{1+Ksv1*\left[O2\right]}+1-f\right),$$ (Eq. 1)

where τ_0_ = 54.8711 μs, *f* = 0.82587 and *K*_*sv*_ = 0.01683 μM^-1^. FLIM and PLIM images, histograms and intensity data were then exported in Microsoft Excel software and used to calculate averaged values. Normalized FLIM histograms were presented as pixel frequencies (as percentage) of a given τ_m_ determined from all separate FLIM images made for the same sample. They were used to calculate cells proliferation rates as areas under the curve and main peak using integration function in Origin 6.0 software (Originlab, USA). The analysis of immunofluorescence images, FLIM-based false color based calculation in RGB ‘blue channel’ (obtained by RGB channel function in Adobe Photoshop software) and intensity-based counting of cell nuclei were performed using ImageJ software (www.fiji.sc).

Unless otherwise stated, all experiments were performed in triplicate (as independent experiments). The experiment on BrdU loading and time-dependent calibration was performed once, with two different BrdU concentrations (25, 100 μM). Averaged fluorescence lifetime distribution histograms corresponding to each loading time were obtained from 5 independent imaging areas of the same samples (~150–200 cells). Statistical analysis of intensity and τ_m_ values for no BrdU /+BrdU cells was done using *t*-test with confidence level p = 0.05. Statistical analysis of APH and metformin effect on intestinal organoids was done using Mann-Whitney U test with confidence level p = 0.05. In figures, N shows a number of microscopy image areas and corresponding distribution histograms used for statistical calculation. Each area contained 150–300 cells and approximately 750–1500 cells were used for analysis in total for each type of calculations.

## Results

### Comparison of FLIM and intensity imaging modes

**Fig 1:**
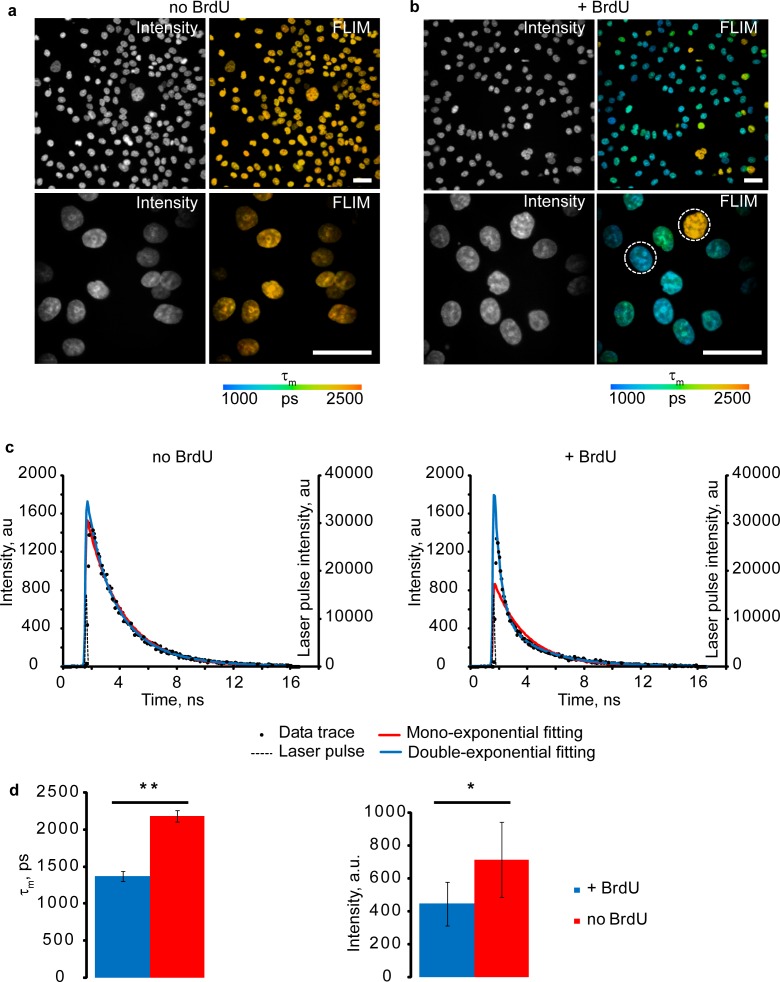
Quenching effect of BrdU on HXT fluorescence. **(a,b)** Images of live synchronized HCT116 cells released from APH block without (a) and with (b) BrdU labeling (100 μM, 4 h), stained with HXT (1 μM, 30 min). Scale bar is 50 μm. **(c)** Representative examples of HXT fluorescence decays (data trace) for individual pixels in selected nuclei (indicated by circles on (b)) showing mono- and double-exponential fittings. **(d)** Average τ_m_ (left) and intensity (right) signals for no BrdU (red, n = 12) and +BrdU (blue, n = 15) nuclei. Asterisks indicate significant difference between groups (p < 0.05): *—p < 0.001, **—p < 0.00001. Error bars show the standard deviation.

The quenching effect of BrdU on fluorescence of dsDNA-bound HXT was studied in live HCT116 human colon cancer cells having cell cycle duration of ~18 h. Cultures were synchronized by aphidicolin (APH) blocking cells at G_1_/S phase, stained with 1 μM HXT for 30 min, exposed to BrdU for 4 h and analyzed by FLIM-TCSPC microscopy under 405 nm excitation, which was non-damaging to the cells. Measurements revealed double-exponential decay profiles of HXT fluorescence, which were strongly influenced by BrdU (Fig 1). For quantification we used mean fluorescence lifetime values (τ_m_) calculated as:

tm=a1*t1+(1−a1)*t2, (Eq. 2)

where a_1_ is the fraction of the first component. Being a characteristic of microenvironment of HXT dye, τ_m_ was seen to provide excellent discrimination between the different BrdU concentrations, incorporation time and physiological conditions.

Using pixel-by-pixel calculation of τ_m_ values in FLIM software, false-color fluorescence lifetime images were generated, which revealed rather homogeneous distribution of τ_m_ in the nuclei, particularly in cells without BrdU (Fig 1). Thus, prior to BrdU exposure average nuclear τ_m_ was 2185 ps ± 77 (N = 12) (Fig 1D). After the exposure most of the cells in synchronized culture reduced their τ_m_ (Fig 1B and 1D) as a result of BrdU incorporation during DNA replication (S phase). Hence, we concluded that average nuclear τ_m_ can be used to quantify the degree of BrdU incorporation in DNA and progression of cell cycle through S phase.

Average intensity counts for no BrdU/ +BrdU nuclei were analyzed and found to be 714±228 and 446±134 photon counts/ per pixel, respectively (the most abundant τ_m_ values for +BrdU nuclei were 1370 ps ±64, N = 15; Fig 1C). Although reflecting the quenching effect of BrdU on HXT [11], nuclear fluorescence intensity values showed high variability and poor analytical performance (Fig 1D).

We also tested mouse embryonic fibroblasts (MEF), which have a slower cell cycle (21–24 h) and observed similar decreases of τ_m_ upon proliferation of asynchronous culture (S1 Fig).

### Analysis of cell proliferation by FLIM

**Fig 2:**
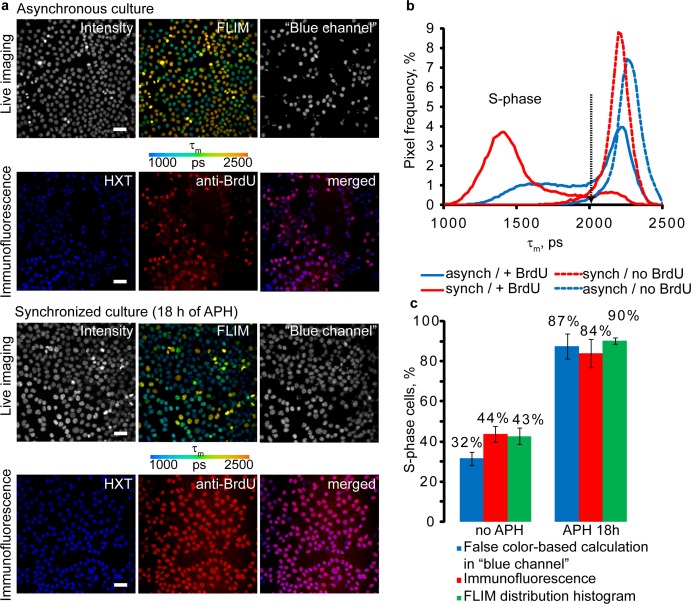
Comparison of FLIM and immunofluorescence methods of cell proliferation analysis. (**a)** Asynchronous and synchronized live HCT116 cells were incubated with BrdU (100 μM, 4 h) and stained with HXT (1 μM, 30 min). Immediately after FLIM cells were fixed with 4% paraformaldehyde and stained with anti-BrdU antibody. Scale bar is 50 μm. **(b)** Average (n = 5) distributions of τ_m_. Black arrow indicates threshold τ_m_, which differentiates between S phase and non S-phase cells. **(c)** Cell proliferation rates calculated by the different methods. Bar chart shows fractions of total cell numbers and standard deviation for +BrdU cells (S-phase). The mean values were calculated from five different images of the asynchronous and synchronized cell cultures.

To better visualize the effect of BrdU and cell division, we plotted frequency distribution histograms of τ_m_ values for pixels of FLIM images. Averaged histograms for several regions of interest (ROI) gave us accurate τ_m_ distributions for each sample. Thus, peaks on these histograms correspond to the most abundant τ_m_ values in samples. Examples of averaged FLIM histograms obtained for asynchronous and synchronized HCT116 cell cultures are shown on Fig 2. They both demonstrate narrow peaks, partially overlapping with one of the peaks for +BrdU samples. Histogram of synchronized +BrdU cells was clearly different from asynchronous +BrdU cells by appearance of pronounced well defined peak with maximum of τ_m_ around 1300–1400 ps. The asynchronous culture showed broad distribution without clear maxima (Fig 2B). Areas under specific regions of the histogram reflect the fractions of cells in S phase of the cell cycle and can be determined by integration function (whole area corresponds to 100% of cells in population). According to “no BrdU” histograms threshold τ_m_ value of 2005 ps was selected for unambiguous discrimination of +BrdU cells. Calculation of cells in S phase using false-color FLIM images can also be performed using split RGB channels, e.g. using widely available software ImageJ. For this an intensity image of ‘blue channel’ (RGB image) showing only nuclei with blue and green colors should be used for intensity-based enumeration of +BrdU cells (Fig 2A). In this case nuclei with transient yellow colors (corresponding to low BrdU loading) are filtered from counting. However, the precision of such analysis is proportional to percentage of dividing cells in population, i.e. in synchronized culture (Fig 2C, false color-based calculation).

To verify the correct identification of S phase positive cells by the new method, we fixed the samples with paraformaldehyde, stained with anti-BrdU antibody (Fig 2A) and analyzed by fluorescence microscopy. We found that both live FLIM and antibody-aided methods gave similar percentage of BrdU-positive cells, both in asynchronous and synchronized cultures: 42.7±4% and 90.1±1.5% for FLIM histograms and 43.8±3.9% and 84.1±7.0% for immunofluorescence methods, respectively (Fig 2C).

### Tracing the cell cycle

We noticed the dose-dependent effect of HXT and BrdU concentrations on τ_m_ (S2 Fig), and found 25 μM BrdU to provide sufficient staining and efficient separation of τ_m_ signals in prolonged (up to 48 h) experiments with cultured cells. Optimal concentration of HXT was 1 μM (S2 Fig). These conditions were used in further experiments.

**Fig 3:**
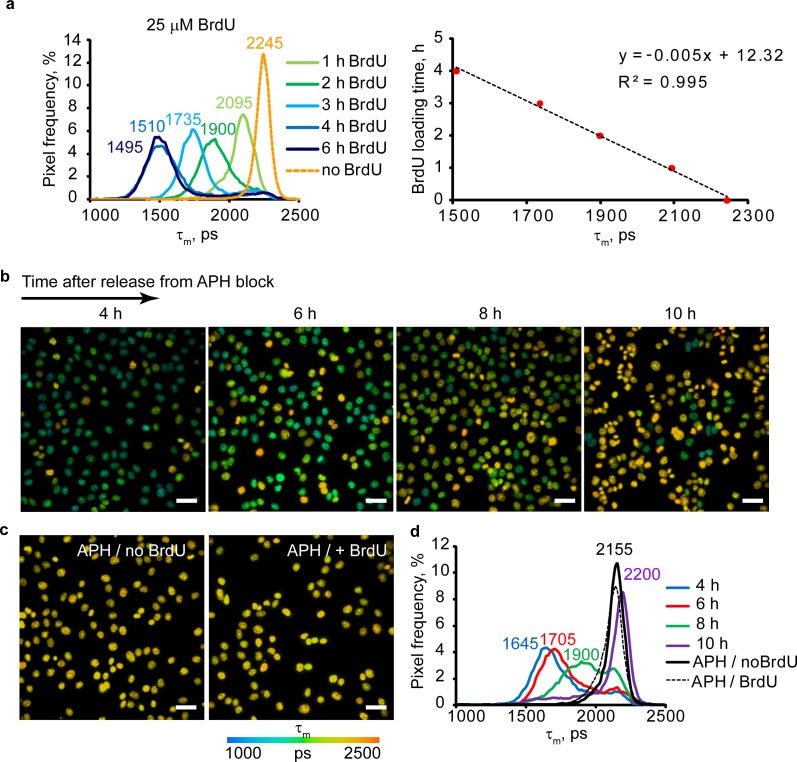
Tracing of cell cycle and duration of S phase in live HCT116 cells. **(a)** τ_m_ histograms for BrdU (25 μM) incorporation at different times (left), and calibration plot for mean fluorescence lifetime, τ_m_ (right, N = 5). **(b, c)** FLIM images of APH-synchronized culture with BrdU incorporation (25 μM) (b), and control synchronized cells with and without BrdU loading (c). Scale bar is 50 μm. **(d)** Average histograms of τ_m_ for images shown in (a) and (b) (N = 5). Note that at 6 h after APH block release cells stop BrdU incorporation and return to “control” conditions. Time points indicate the time of imaging.

Using aphidicolin (APH)-synchronized HCT116 cells exposed to 25 μM BrdU 30 min after the release of block, we analyzed τ_m_ distribution during 6 h period (Fig 3A). The value of τ_m_ at 0 h was determined for BrdU-non-loaded cells. The same analysis was repeated for 100 μM BrdU loading (S2 Fig). Plotting the most abundant average τ_m_ at each time point (corresponding to main peaks on distribution histograms), we observed its linear dependence on loading time, τ_s_ (Fig 3A, S2 Fig), which can be used for quantification of S phase duration for cell populations (for 25 μM BrdU):

ts=−0.005*tm+12.32, (Eq. 3)

To confirm this, we also conducted a cell cycle tracing experiment in which synchronized HCT116 cells were exposed to 25 μM BrdU for 4 h at different time points (0, 2, 4 and 6 h) after the release of APH block (Fig 3B). The main peak values on τ_m_ histograms (Fig 3B–3D) were used to calculate the time of BrdU uptake (from the obtained linear equation according to Eq 3, (Fig 3A). We found that cells entered S phase predominantly after the release of APH block and replicated their DNA for 6.3±0.7 h after the release of APH block. FLIM images also confirmed that after 6 h most of cells stopped accumulating the BrdU (Fig 3B, 10 h point). Histogram peaks (Fig 3D) for APH/no BrdU- and APH/+BrdU cells (Fig 3C) overlap, indicating that no BrdU uptake occurred in the presence of APH.

We also applied FLIM to trace labeled cells through several division cycles. Synchronized cells loaded with 25 μM BrdU for 4 h were imaged on the day (S_0_ and post-mitotic M_0_ phase), then 24 h (first cell division, S phase D_1_, S_1_) and 48 h later (second cell division, S phase D_2_, S_2_). Control cells from the same passage were also grown and analyzed by FLIM. After the imaging, cells were trypsinized and counted. S2 Fig shows that increases in cell numbers correlated with the shifts in τ_m_ values towards control peak (no BrdU). It was possible to reliably trace BrdU-labeled cells during two division cycles, and after this (at D_2_ stage) nuclear BrdU was diluted to undetectable levels.

### Visualizing cell proliferation in tumor spheroids

**Fig 4:**
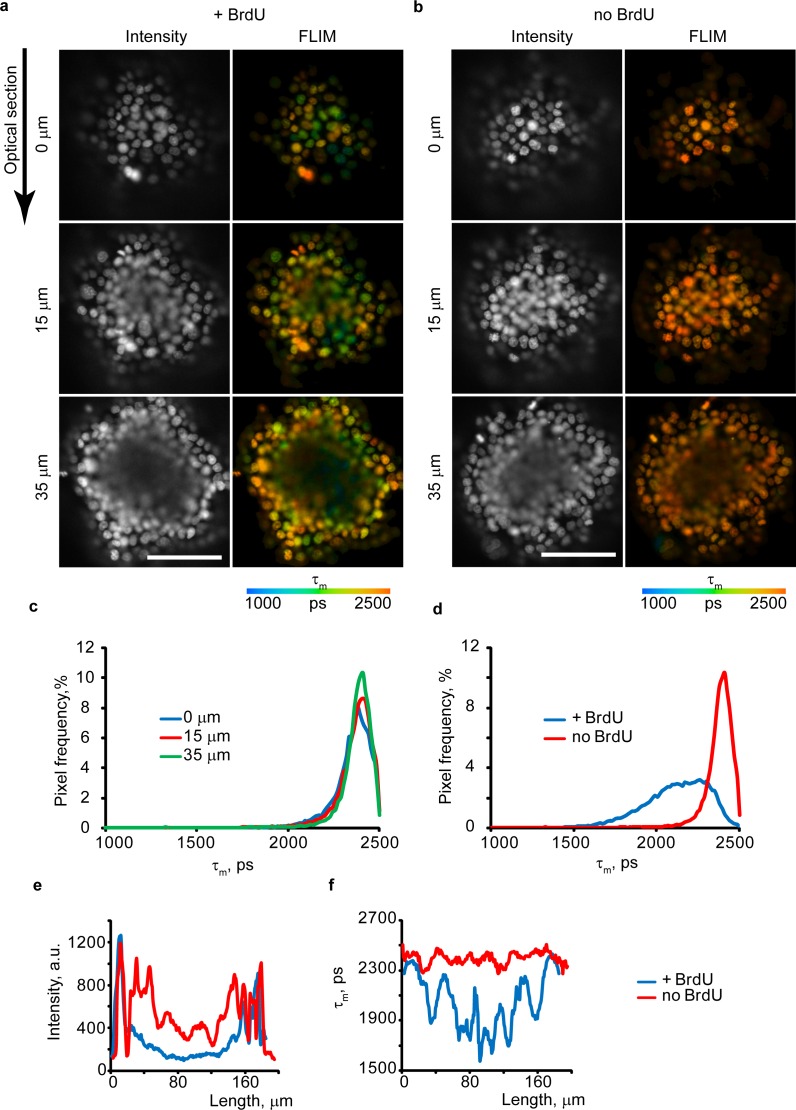
FLIM imaging of live tumor spheroids from HCT116 cells. **(a,b)** Confocal optical sections for spheroids loaded or unloaded with BrdU (100 μM, 4 h), collected at different depths. Fluorescence intensity is shown in grayscale. Scale bar is 100 μm. **(c, d)** τ_m_ histograms for different optical sections (depths 0–35 μm) for no BrdU (c) and +BrdU (35 μm depth, d) spheroids. **(e,f)** Comparison of the Intensity (e) and τ_m_ (f) profiles across the spheroid (35 μm depth). Representative images are shown. N = 3.

Adherent 2D cell cultures are convenient for live cell fluorescence microscopy and immunostaining, but they normally lack physiological context, such as cell morphology, cell-cell interactions, gradients of nutrients and other factors [28, 29]. 3D tissue models such as tumor spheroids [24, 30] are more relevant, but bring new technical challenges [23]. Using paraformaldehyde fixation and immunostaining of BrdU-labeled cells, we observed preferential labeling of spheroid periphery and very weak of the core, hardly distinguishable from autofluorescence (S3 Fig). This result can be interpreted wrongly as there are more dividing cells at the periphery than in core of spheroids. The poor in-depth staining of spheroids by antibodies (and many other common fluorescent probes) due to reduced diffusion across spheroids contrasted with their fast and efficient staining by both HXT and BrdU (Fig 4A and 4B).

Confocal FLIM showed efficient staining of spheroids of >200 μm in size with HXT, which also produced uniform τ_m_ values at different depths, without any side effects and measurement artifacts (e.g. attenuated intensity signals due to light scattering) (Fig 4). Exposure of spheroids to BrdU for 4 h produced nuclei with low τ_m_ (+BrdU), less at the periphery and more in the core, as evidenced by FLIM histograms (Fig 4A, 4D and 4F). Line profiles of fluorescence intensity and lifetime signals across the +BrdU/no BrdU spheroids shown in Fig 4E and 4F (35 μm depth) demonstrate that intensity signals are largely reduced in the core compared to the periphery due to depth-dependent light penetration, which makes this parameter unreliable for monitoring BrdU incorporation. Conversely, τ_m_ values were unaffected by depth and the number of detected photons was sufficient for accurate FLIM (Fig 4C and 4F). Thus, the method is also applicable to live tumor cell spheroids and 3D tissue models, to visualize proliferating cells, both at periphery and in deep core regions. Light penetration depth can be further increased by two-photon excited or light-sheet microscopies [23].

### Action of metformin on proliferation of epithelial cells in intestinal organoids

Metformin is an anti-diabetic drug associated with reduced risk of cancer [31]. Its pharmacological effects are linked to inhibition of cell proliferation: indirectly, via AMPK activation (downregulates cyclin D1 and inhibits cells in G_1_/S phase [32] in response to mitochondrial complex I inhibition) and directly, via p53-dependent activation of REDD1 (a negative regulator of mTOR), which leads to cell cycle arrest [33]. These effects were demonstrated in various cancer cell lines and tumor tissues. However, there is no evidence that metformin works in the same way in normal tissue such as intestinal mucosa where it can accumulate at high levels [34, 35].

We analyzed the effect of metformin on cell proliferation in epithelia of mouse intestinal organoids. Intestinal organoids are complex 3D cultures produced from primary stem cells, which have the characteristic villi-crypt organization, with epithelial monolayer composed of different cell types around lumen. The epithelium contains stem and Paneth cell niches at the bottom of crypts, amplification zones with actively proliferating undifferentiated cells and lineages of main differentiated cells: enterocytes, goblet cells and enteroendocrine cells [36].

**Fig 5:**
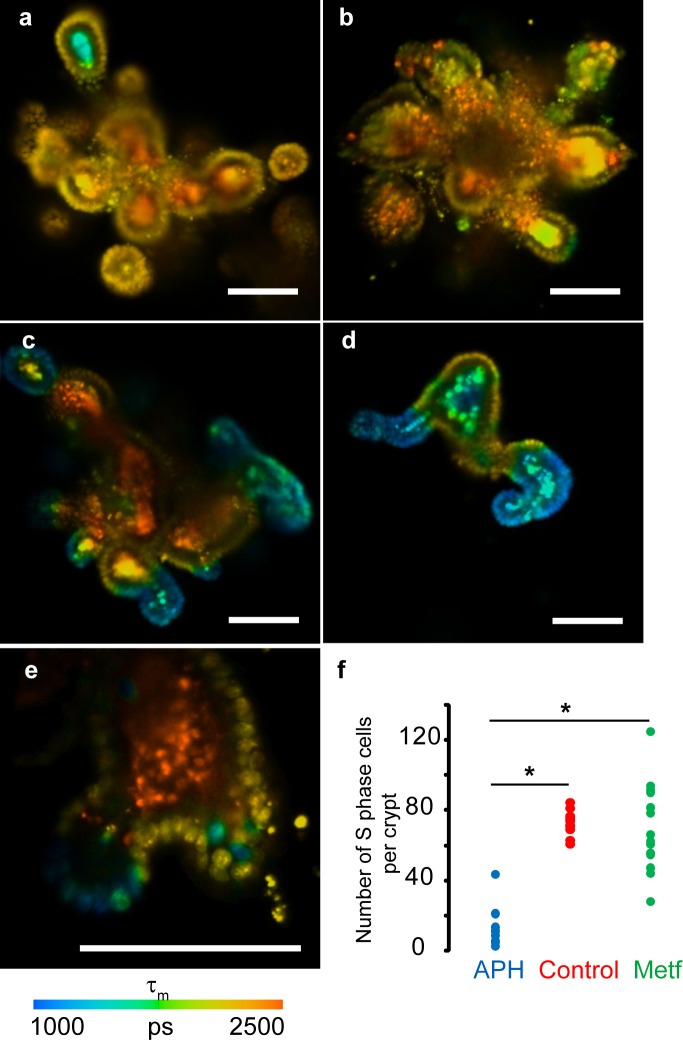
FLIM imaging of live mouse intestinal organoids stained with HXT (1.5 μM, 4 h) and BrdU. **(a)** Control organoid (no drugs, no BrdU) shows homogenous τ_m_ distribution in epithelial monolayer and heterogeneous τ_m_ in lumen regions. (**b**) Organoid treated with APH and BrdU (100 μM, 18 h). **(c,d)** Heterogeneity of organoids (metformin group). (**e**) FLIM image of an individual crypt from organoid incubated with BrdU (100 μM, 4 h) shows fewer nuclei with decreased τ_m_. (**f**) Effects of APH and metformin compared to non-treated culture. (N = 9 (APH), N = 12 (control), N = 16 (metformin). N corresponds to a number of organoids in each group. Asterisks indicate significant difference between groups (p < 0.05). Scale bar is 100 μm.

Upon incubation with BrdU only actively dividing stem cells and cells from the amplification zone, but not the differentiated non-dividing cells, should take up BrdU and thus decrease their τ_m_. To prove this, we incubated intestinal organoids cultured in Matrigel matrix with 0/ 100 μM BrdU for 4 and 18 h, stained them with HXT and analyzed by FLIM (Fig 5). Indeed, organoids without BrdU showed homogeneous HXT staining of cell monolayer with τ_m_ = 2220 ± 38 ps (Fig 5A). In contrast, organoids loaded with BrdU for 18 h had defined zones in cell monolayer with significantly reduced τ_m_ (Fig 5C and 5D), which correspond to amplification zones of intestinal epithelia. Organoids loaded with BrdU for shorter periods (4 h) had a lower number of nuclei with reduced τ_m_ (Fig 5E). For further experiments we used 18 h incubation with BrdU.

It is worth noting that organoid cultures showed bright autofluorescence of the lumen with long τ_m_ ranging in 1500–2500 ps (Fig 5, S4 Fig), which complicated FLIM imaging, generation of histograms and calculation of proliferation rates. Co-staining with CellTox Green revealed that HXT-stained only live cells in organoid epithelial layer (S4 Fig). Imaging of organoids under the same acquisition settings, without HXT but with cell monolayer stained with red-emitting tetramethylrhodamine methyl ester (TMRM, marker of polarized mitochondria) proved that cell monolayer shows only minor autofluorescence, while in the lumen it was bright and had broad spectrum (S4 Fig). Nevertheless, HXT intensity signals in the epithelia were comparable with lumen autofluorescence, and their τ_m_ allowed reliable discrimination (S4 Fig). Since organoid autofluorescence had τ_m_ values of >1500 ps, we used the range 1000–1510 ps for the enumeration of +BrdU nuclei.

We found that even when derived from the same batch of primary cells, organoids were very heterogeneous in size, number of crypts and activity of proliferation zones (Fig 5C–5D). For analysis we normalized the total number of proliferating cells in each organoid (area of the histogram under τ_m_ of 1000–1510 ps) for the number of crypts (finger-like protrusion structures with amplification zone). These values were calculated for all imaged organoids in metformin-treated groups and analyzed statistically. Control samples represented group without metformin treatment and with 24 h treatment with 0.5 μg/ml APH. Experimental group of organoids was incubated with metformin (500 μM) in growth media for 3 days. As expected, APH-treated organoids showed significantly lower level of cell proliferation (Fig 5B and 5F) compared to metformin-treated group and untreated control (p < 0.05). Control and metformin groups both showed high proliferation rate (Fig 5F), suggesting that metformin did not significantly influence the cell proliferation in intestinal organoids. This agrees with reported minor side effects of metformin therapy (gastrointestinal irritation, diarrhea, clamps, vomiting and nausea) which can be avoided by gradually increasing the dose to therapeutic levels [37].

### Multi-parametric imaging of intestinal organoids

**Fig 6:**
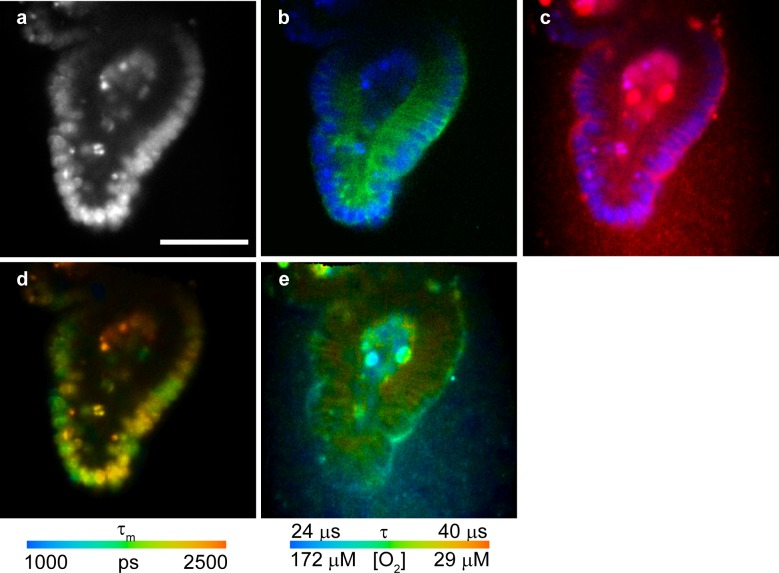
Multi-parametric FLIM imaging of mouse intestinal organoids. **(a,b,c)** Intensity images of HXT (a), HXT (blue) merged with lipid raft stain (green) (b), and HXT (blue) merged with cell-penetrating O_2_-sensitive probe Pt-Glc (red) (c). **(d)** HXT τ_m_ image informing on cell proliferation (405 nm exc., 438–458 nm em.). **(e)** τ_m_ of Pt-Glc (405 nm exc., 635–675 nm em.) informing on cell/tissue oxygenation. Scale bar is 50 μm.

Detection of dividing cells by FLIM allows incorporation of this assay in multi-parametric imaging of live 3D tissue models, to monitor relevant physiological parameters. Combined FLIM-PLIM with multiplexing in spectral and time domains (ns and μs probes) allows imaging of oxygenation, division and localization of proliferating cells, Ca^2+^ fluxes and cell death (necrosis, apoptosis) [15, 17, 38]. We performed multiplexed FLIM-PLIM of intestinal organoids loaded with BrdU for 4 h and then co-stained with HXT, O_2_-sensitive phosphorescent probe Pt-Glc [18] and Cholera Toxin-Alexa Fluor 488 (CTX) conjugate (lipid raft-specific probe). HXT and Pt-Glc are excitable at 405 nm, but have different emission spectra (blue and red, respectively) and lifetime ranges: 1–3 ns for HXT and 20–60 μs for Pt-Glc. Alexa Fluor 488 is spectrally distinct from HXT and Pt-Glc. Fig 6 demonstrates how multiplexed spectral and lifetime imaging can inform on the O_2_ distribution within organoid, activity of proliferating cells and their specific localization.

## Discussion

The new FLIM method based on HXT quenching by incorporated BrdU allows monitoring of cell proliferation in live cultures. Compared to fluorescence intensity readout, FLIM is largely independent on dye concentration and depth of confocal scanning, which is important for 3D cell and tissue models. Since both HXT and BrdU easily penetrate multiple cell layers, and corresponding staining procedures are well established, the method is broadly applicable to various cell types and complex multicellular structures including spheroids, organoids, primary tissue sections and biopsies. This is in contrast with the use of genetically encoded fluorescent proteins, which need transfection or use of transgenic cell lines [6, 7].

FLIM of proliferating cells can be combined with analysis of other important biomarkers, such as cell oxygenation, NAD(P)H and FAD, transmembrane ion fluxes, mitochondria polarization, and provide multi-parametric high-content readout. Experimental procedure is simple and sample preparation time is short, compared to immunostaining and EdU detection. It also avoids potential artifacts due to cell fixation and/or fluorescence intensity readout [39].

Different cell models might have different sensitivity to BrdU and HXT concentrations even if generally these compounds have low toxicity. We found that 100 μM BrdU concentration recommended for cell labeling in tracing experiments partly inhibits proliferation of HCT116 cells (data not shown), but can be optimized for prolonged (up to several days) experiments. On the other hand, with the calibration experiments (S2 Fig) it is possible to use τ_m_ to discriminate between different BrdU concentrations and incorporation times, thus seeing the effect of various physiological conditions on the cell cycle. In contrast to previous reports [40, 41], we did not see major effects of Hoechst 33342 on cell cycle duration or significant phototoxicity upon FLIM imaging (S5 and S6 Figs). The latter can be explained by much lower laser powers employed in TCSPC-FLIM method [15], broadening its applicability to live cell imaging.

Accordingly to our results, the quenching effect of BrdU was largely independent on the state of chromatin condensation: in asynchronous cell culture we observed uniform distribution of quenched fluorescence lifetimes and linear dependence of τ_m_ from BrdU loading (S2 Fig). In addition, long (16 h) loading with BrdU resulted in presence of both S and M phase cells, having very similarly quenched fluorescence lifetimes (not shown).

Method adaptation to a new cell model requires initial optimization, calibration and appropriate controls. In addition, cell cultures are heterogeneous in cell cycle duration and ability to proliferate, which can be influenced by passage number. Even synchronized cultures showed rather broad distribution of S phase duration. For HCT116 cells, average duration of S phase was ~ 6 ± 0.7 h (determined with APH block), as compared to 8–9 h reported in the literature [42]. The difference could be due to the different synchronization method, cell density and passage number used. Therefore, for best accuracy of the FLIM assay and quantification of S phase duration, τ_m_ calibration should be performed on the same passage and cell density as the main experiment. Using different synchronization methods and separating other stages of cell cycle, the full cell cycle can be traced. We also tried to see the upper limits of the monitoring of the cell cycle (S2 Fig) and found that effect of BrdU becomes undetectable after second division (D2). However it should be kept in mind that this is caused by sensitivity of our chosen dye (HXT) and its quenching by BrdU. It is possible that other dsDNA staining dye can be used in described assay with better performance.

Spectral properties of HXT are not optimal for some 3D tissue models (i.e. spheroids of > 200 μm size or intestinal organoids, having strong luminal autofluorescence) and can be also influenced by limited light penetration depth, diffraction and light scattering. Still, even with non-ideal resolution, we were able to show that FLIM method allows identifying proliferating cells inside live spheroids better than staining with antibodies of whole spheroids (Fig 4, S3 Fig). Characterization and filtering out of autofluorescence lifetimes by using distribution histograms (S4 Fig, Fig 5) allowed to efficiently study the pharmacological effect of metformin in mouse intestinal organoids. We envision that the performance of the method can be improved by using two-photon excitation, light-sheet microscopy and by development of longwave fluorescent DNA stains and BrdU analogs. An increased sensitivity of fluorescent probe to BrdU can indeed allow the decrease of labeling time, which can improve method accuracy, and preparation time. Overall, described method opens up new uses of fluorescent DNA stains and a new set of applications for live cell FLIM. It also adds another important parameter to quantitative and multi-parametric imaging techniques of complex cell and tissue cultures.

## Supporting Information

S1 Fig Live cell FLIM of HXT-stained nuclei in MEF cells. Cells were loaded with BrdU (100 μM, 18 h) and counter-stained with HXT before imaging. Scale bar is 10 μm. N = 3.
(TIF)

S2 Fig Optimization of BrdU and HXT loading concentrations for FLIM in HCT116 cells. **(a)** The effect of HXT loading concentration (0.5–4 μM, 30 min) on τ_m_ (N = 3). The concentrations 0.5 μM and 1 μM, overlapping and showing maximal τ_m_ values were chosen. **(b)** Concentration-dependent effect of BrdU loading on τ_m_. HXT (1 μM) and BrdU (5–100 μM, 4 h) were used. **(c)** Time-dependent effect of BrdU loading on τ_m_. Synchronized cells having the same passage and density were incubated with BrdU (100 μM, 1–6 h), after release from APH block (30 min post-release). (N = 5). Right: the linear dependence of BrdU loading time from τ_m_. **(d)** Tracing of BrdU-positive cells in cell culture during several rounds of cell divisions. Averaged FLIM histograms (N = 5) for different stages of experiment are presented. Synchronized HCT116 cells were loaded with BrdU (25 μM, 4 h) during first S phase (S0) after release from APH block and imaged immediately. 24 h (D1) and 48 h (D2) cells were imaged again. The control sample (no BrdU D2) represents synchronized cells without loading with BrdU, cultured in parallel and imaged together with D2 experimental sample. After all stages of the imaging, cells were collected and counted to determine the average cell number at different stages of experiment, as shown in the table. N shows a number of images used for analysis. Each image contained approximately 200 cells.
(TIF)

S3 Fig Immunofluorescence of BrdU-loaded nuclei in HCT116 spheroids. Three separate confocal sections are shown with fluorescence of HXT (blue) and antibody-stained BrdU (red). Scale bar is 100 μm. N = 4.
(TIF)

S4 Fig Intestinal organoids display strong luminal autofluorescence. **(a)** Comparison of fluorescence of TMRM (20 nM, exc. 540 nm, em. 565–605 nm) with autofluorescence of lumen (exc. 405 nm/ em. 438–458 nm). The emission range of 438–458 nm did not show significant autofluorescence for cell monolayer (labeled with TMRM); however the autofluorescence signals from lumen were present in both 438–458 nm and 565–605 nm emission channels. **(b)** Comparison of fluorescence of CellTox Green (labels dying cells, exc. 488 nm, em. 512–536 nm) with HXT (exc. 405 nm, 438–458 nm) reveals that lumen does not contain significant amount of dead cells. **(c)** Average fluorescence intensity signals of HXT at the cell layer and in lumen, contrasted with autofluorescence. Error bars represent the standard deviation. Scale bar is 100 μm.
(TIF)

S5 Fig The effect of HXT staining on cell cycle. Live HCT116 cells were stained with HXT (1.5 μM, 30 min) or remained untreated (no HXT). 6 h post-treatment, cells were pulsed with BrdU (100 μM, 30 min), fixed and immunostained with anti-BrdU antibody. The percentage of BrdU-positive (S-phase cells) was calculated for each group and analyzed by *t*-test (p = 0.05). **(a)** Immunofluorescence images. Scale bar is 50 μm. **(b)** Bar charts of the mean percentage of cells in S-phase between the control and experimental groups. N = 4. Approximately 1300–1400 cells were analyzed in each group.
(TIF)

S6 Fig Evaluation of HXT phototoxicity with live HCT116 cells. The live cells were loaded with HXT (1.5 μM, 30 min), washed and illuminated with 405 nm BDL-SMC (< 1 mW power) laser (1 min, + 9 min break, 10 repeats). 30 min post-illumination, cells were stained with CellTox Green (0.1%, 5 min) and analyzed for viability. Control group was illuminated only in order to count total cell number and damaged cells. **(a)** Transmission light images **(b)** Confocal microscopy images stained cells. **(c)** Average numbers of dead cells between the groups. Data were evaluated using *t*-test with confidence level of p = 0.05. (N = 2). Approx. 1100 cells were analyzed.
(TIF)
